# Superconducting phase diagram of finite-layer nickelates Nd_*n*+1_Ni_*n*_O_2*n*+2_

**DOI:** 10.1038/s41535-025-00786-z

**Published:** 2025-07-01

**Authors:** Andreas Hausoel, Simone Di Cataldo, Motoharu Kitatani, Oleg Janson, Karsten Held

**Affiliations:** 1 Institute for Theoretical Solid State Physics, Leibniz Institute for Solid State and Materials Research Dresden, Dresden, Germany; 2 Dipartimento di Fisica, Sapienza Università di Roma, Roma, Italy; 3 Department of Material Science, University of Hyogo, Ako, Hyogo, Japan; 4 Institute of Solid State Physics, TU Wien, Vienna, Austria

**Keywords:** Superconducting properties and materials, Electronic properties and materials

## Abstract

Following the successful prediction of the superconducting phase diagram for infinite-layer nickelates, here we calculate the superconducting *T*_c_ vs. the number of layers *n* for finite-layer nickelates using the dynamical vertex approximation. To this end, we start with density functional theory, and include local correlations non-perturbatively by dynamical mean-field theory for *n* = 2–7. For all *n*, the Ni ${d}_{{x}^{2}-{y}^{2}}$ orbital crosses the Fermi level, but for *n* > 4 there are additional (*π*, *π*) pockets or tubes that slightly enhance the layer-averaged hole doping of the ${d}_{{x}^{2}-{y}^{2}}$ orbitals beyond the leading 1/*n* contribution stemming from the valence electron count. We finally calculate *T*_c_ for the single-orbital ${d}_{{x}^{2}-{y}^{2}}$ Hubbard model by dynamical vertex approximation.

## Introduction

The discovery of superconductivity in nickelates by Li, Hwang et al.^1^ has opened a new and very active area of research. In fact, superconducting nickelates remain a largely unexplored family of strongly-correlated systems, and offer researchers a unique and novel testbed to study unconventional superconductivity.

However, synthesizing infinite-layer nickelates (Ca,Sr)_*x*_*R*_1−*x*_NiO_2_ with rare earth *R* = Nd, La, Pr, Sm that are actually superconducting has proved to be challenging, and only a few groups succeeded^2–14^. The primary obstacle to synthesize superconducting nickelate films and bulk materials is the large degree of disorder, stacking faults and off-stoichiometry. This is caused by the complicated synthesis process which involves first growing (Ca,Sr)_*x*_*R*_1−*x*_NiO_3_ and then reducing it to (Ca,Sr)_*x*_*R*_1−*x*_NiO_2_ with CaH_2_ or H^13,15^. The necessity to hole-dope the system into the superconducting state with Ca (or Sr) increases the chances of disorder. This, in combination with the oxygen reduction, prevents synthesizing bulk nickelates with *x* > 8%^16^. It is not surprising that, even among the superconducting films, there is a large variance in the measured properties. Cleaner films show resistivities lower by a factor of three, while at the same time, the critical temperature *T*_*c*_ is considerably larger^9,10^.

**Fig. 1:**
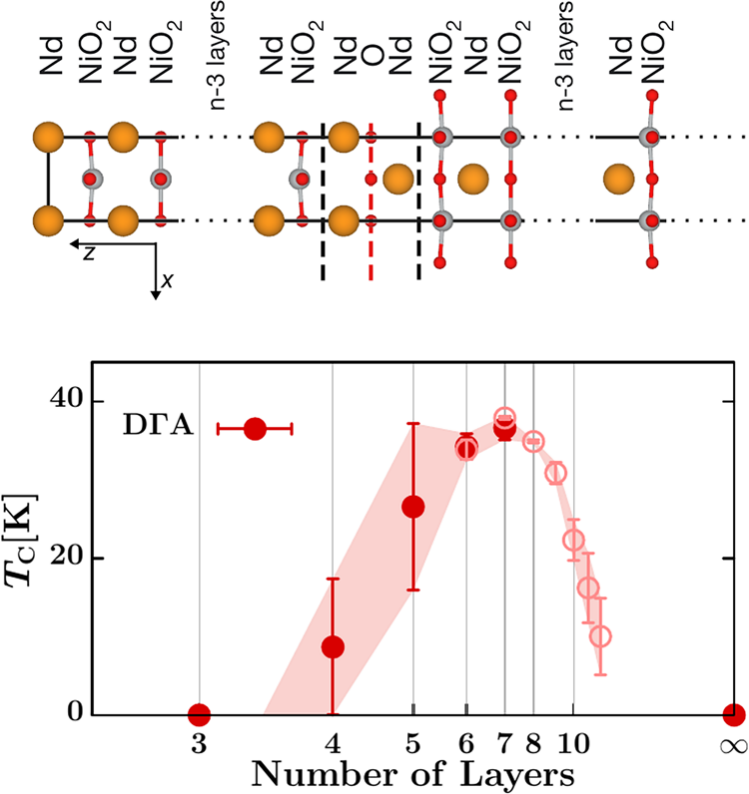
Phase diagram of finite-layer nickelates Nd_*n*+1_Ni_*n*_O_2*n*+2_. Top: Crystal structure of finite-layer nickelates Nd_*n*+1_Ni_*n*_O_2*n*+2_. Nd, Ni, and O atoms are indicated as large orange, medium gray, and small red spheres, respectively. Dashed black lines indicate the Nd–O–Nd stacking fault. Bottom: Phase diagram superconducting *T*_*c*_ vs. number of layers *n* as calculated by D*Γ*A. The error bar reflects the uncertainty in the hole doping of the ${d}_{{x}^{2}-{y}^{2}}$ orbital, as discussed in Section “Dynamical vertex approximation”; the open circle for *n*≥6 indicates that we here used an infinite-layer calculation with total doping 1/*n* to mimic the finite layer nickelate.

Against this background, growing finite-layer nickelate films Nd_*n*+1_Ni_*n*_O_2*n*+2_, see Fig. 1 (top), offers a welcome alternative to doping infinite-layer nickelates with Sr or Ca, eliminating at least Sr/Ca disorder. This second family of nickelates presents a series of finite slabs separated by a stacking fault (black dashed lines in Fig. 1) that is delineated by an additional O layer and a shift by half-of-the-unit-cell diagonal perpendicular to the stacking fault. These finite-layer nickelates are a generalization of infinite-layer ones that are recovered for *n* → *∞*. These finite-layer nickelates are not to be confused with the Ruddlesden-Popper series La_*n*+1_Ni_*n*_O_3*n*+1_^17^ whose Ni 3*d* shells are filled with $(7+\frac{1}{n})$ electrons, i.e., between 7 and 8, instead of $(9-\frac{1}{n})$ for the finite-layer nickelates considered here.

Pan, Mundy et al. established the presence of superconductivity in finite-layer nickelates for *n* = 5^6^ and reported ongoing work for other *n*^18^. Similar layered compounds can also form with copper, such as Tl_2_Ba_2_Ca_*n*−1_Cu_*n*_O_2*n*+4_^19^ and HgBa_2_Ca_*n*−1_Cu_*n*_O_2*n*+2_^20^, in which the *T*_*c*_ is typically maximal for *n* = 3. In a fashion similar to cuprates, it is likely that finite-layer nickelates are far from having reached the optimum *T*_*c*_, as both the number of layers and sample quality can still be optimized.

Moving on to the theoretical studies, superconductivity in bulk nickelates^21^ and nickelate heterostructures^22^ has been conjectured long before samples were successfully synthesized. Only after their synthesis in 2019^1^, however, were they thoroughly examined on the theoretical side, turning them into the *hot* topic they currently are^23–33^.

The arguably simplest description for 3*d*^9^ nickelates is that of a one-band Hubbard model plus largely detached pockets originating from the Nd 5*d* orbitals^34,35^. This simple description was recently confirmed by angular-resolved photoemission spectroscopy (ARPES) that only showed a Ni 3${d}_{{x}^{2}-{y}^{2}}$ Fermi surface and a pocket at $\left(\pi ,\pi ,\pi \right)$ (“A pocket”) for Sr_*x*_La_1−*x*_NiO_2_^36,37^, in excellent agreement with a priori density functional theory (DFT) plus dynamical mean-field theory (DMFT) calculations^34,38^. Even the superconducting dome determined with the dynamical vertex approximation (D*Γ*A)^34^ well agrees with *a posteriori* experiments^9^, as does the resonant inelastic X-ray spectrum^39,40^. Finite-layer nickelates have been also studied theoretically^41,42^, though hitherto with a focus on the pentalayer found in the first experiment^6^.

In this letter, we calculated the *T*_*c*_ vs. number of layers *n* phase diagram for finite-layer neodymium nickelates on a neodymium gallium oxide substrate. We employed DFT to determine the crystal structure of such strained superlattices and calculate their electronic structure. From there, we included local correlations using DMFT in Ni 3*d* and Nd 5*d* shells, and finally mapped the result to the minimal single-band model to estimate the superconducting *T*_c_ by D*Γ*A. This brings us to the central result of our work—the phase diagram in Fig. 1, showing the dome-like behavior of the superconducting *T*_*c*_ vs. an increasing (from left to right) number of layers *n*. Superconductivity is found for a similar number of layers as in experiment (*n* = 4–8^18^).

## Results

### Density functional theory

The crystal structures for the Nd_*n*+1_Ni_*n*_O_2*n*+2_ series were constructed starting from a supercell of *n* infinite-layer unit cells and adding the stacking fault. The in-plane lattice parameters were constrained to those of cubic NdGaO_3_ (NGO), i.e., *a* = *b* = 3.83 Å, which experimentally is used as a substrate^6,18^, while the *c* axis was relaxed as in ref. ^43^, see Supplementary Note 2.

There is an out-of-plane distortion of the Ni-O bonds, clearly visible in the true-to-scale Fig. 1 (top), which is particularly strong for the layers facing the stacking fault (4°), and rapidly fades in further layers. See Supplementary Note 2 for details on the distortion angles and further information on the DFT relaxation; and Section IV for the methods and codes used. The interaction with the stacking fault causes a substantial difference between *outer* (close to the stacking fault) and *inner* (away from the stacking fault) Ni-O layers. This interaction also introduces a symmetry inequivalence between odd-*n* and even-*n* cases, for which *n* = 2 represents a further special case in which only outer layers are present.

**Fig. 2:**
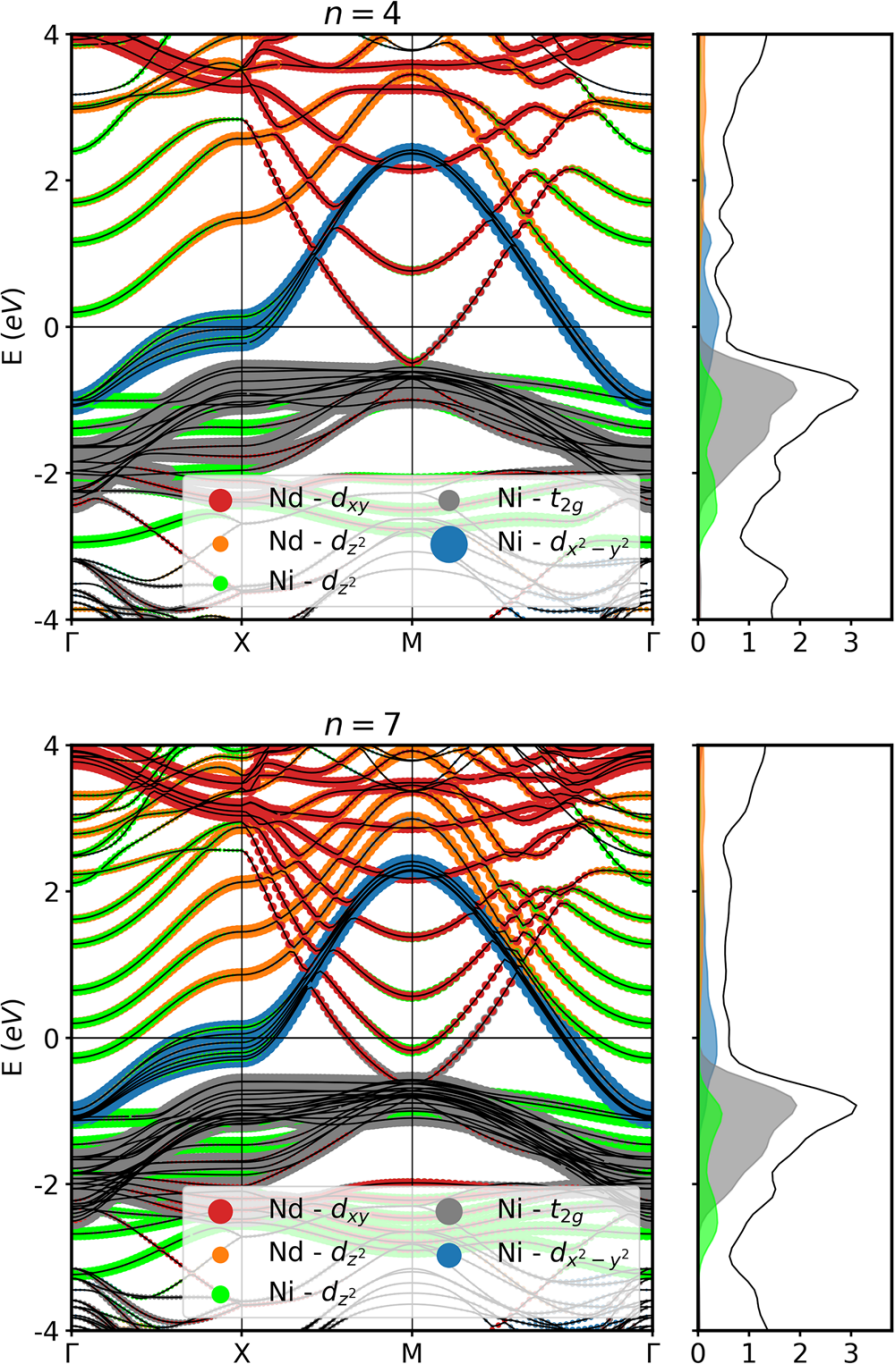
DFT orbital-resolved band structure of Nd_5_Ni_4_O_10_ (top) and Nd_8_Ni_7_O_16_ (bottom). In the right panels the corresponding DFT DOS is shown; see Supplementary Note 3 for *n* = 2, 3, 5, 6 and *∞* layers. The bands are colored with the projection onto the atomic orbitals. Red, gray, orange, blue, and green indicate Nd *d*_*xy*_, Ni t_2*g*_, Nd ${d}_{{z}^{2}}$, Ni ${d}_{{x}^{2}-{y}^{2}}$, and Ni ${d}_{{z}^{2}}$ orbitals, respectively. The DOS is indicated in units of states/eV/Ni; projections onto atomic orbitals follow the same color coding as the bands.

In Fig. 2, we report the DFT-calculated band structure and density of states (DOS), projected onto the most relevant atomic orbitals for *n* = 4 and *n* = 7 layers (for the other values of *n* we refer the reader to the Supplementary Note 3). For both *n*’s, bands of Ni ${d}_{{x}^{2}-{y}^{2}}$ and Nd *d*_*xy*_ character cross the Fermi energy. The latter forms the A pocket of the infinite-layer nickelate which becomes one or several M–A tubes for the finite-layer compound, see Supplementary Note 3. Without the stacking fault, we would have just the infinite-layer structure, but with a backfolding of *n* *k*_*z*_’s into *n* vineyard-like bands for a supercell of *n* layers. We see this general pattern, but the stacking fault also leads to marked differences, particularly for small *n*. Most noteworthy, the electron pocket at the *Γ* pocket formed by Nd with some (admixed) Ni ${d}_{{z}^{2}}$ character, which is still present for *n* = 7, gets lifted above the Fermi energy for *n* = 4. The projected DOS in Fig. 2 (left) also shows predominantly Ni ${d}_{{x}^{2}-{y}^{2}}$ character at the Fermi level, with Ni *t*_2*g*_ and ${d}_{{z}^{2}}$ bands further below and Nd bands above. Changes of the DOS are quite minute when changing the number of layers *n*.

### Dynamical mean-field theory

Due to the partially occupied 3*d* states, specifically the roughly half-filled Ni 3${d}_{{x}^{2}-{y}^{2}}$ orbital, electronic correlations beyond DFT are important, and we take these into account, on a first level, by DMFT^44,45^. To this end, we perform Wannier projections of the band structure onto a model of Ni 3*d* and Nd 5*d* states. Next, we supplement the Wannier-projected Hamiltonian by a local intra-orbital Coulomb interaction of *U* = 4.4 eV (2.5 eV) and a Hund’s exchange *J* = 0.65 eV (0.25 eV) for the five Ni (Nd) 3*d* (5*d*) orbitals, as calculated previously in constrained random phase approximation (cRPA)^33,34^ and used throughout our work^34,40,41,43^. For details of the Wannier projection and the hopping parameters, see the Supplementary Note 2.

Constructing accurate Wannier projections for large-*n* superlattices becomes very cumbersome. Yet, such models are also dispensable, because in this limit the physics of finite-layer nickelates hardly differs from infinite-layer NdNiO_2_. Hence, for *n* ≥ 8, we restrict ourselves to five–plus–five DMFT infinite-layer calculations for one formula unit and a total filling of (9 − 1/*n*) of all ten orbitals. These “infinite-layer” results are indicated in Fig. 1 (above) and Fig. 4 (below) through open circles and continue those of the actual finite-layer calculations quite smoothly with only a minor kink.

**Fig. 3:**
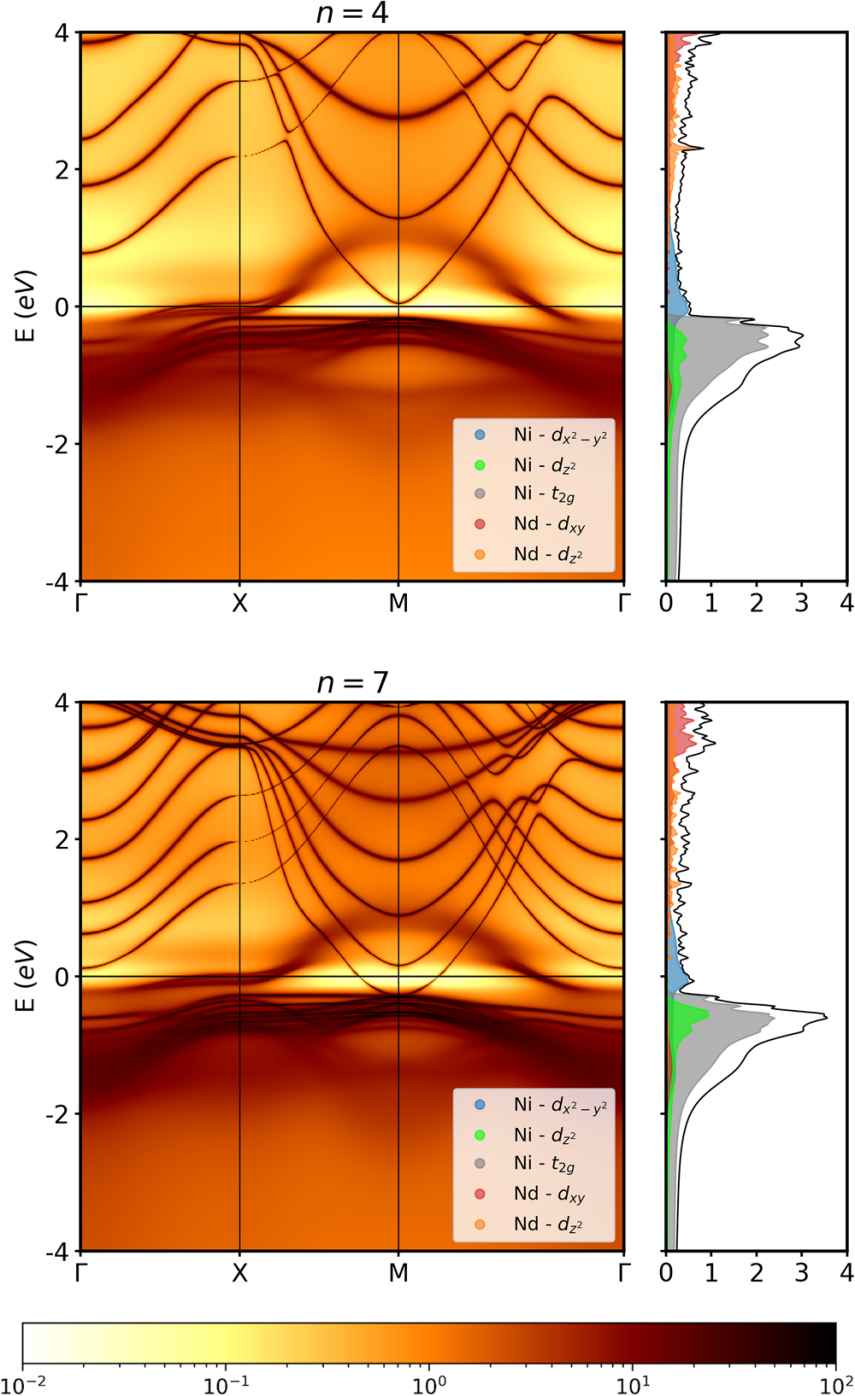
DMFT spectral functions of Nd_5_Ni_4_O_10_ (top) and Nd_8_Ni_7_O_16_ (bottom). In the right panels the corresponding DMFT DOS is shown; calculations are at room temperature; see Supplementary Note 4 for *n* = 2, 3, 5, 6, *∞* layers.

Figure 3 shows the DMFT **k**-resolved and **k**-integrated spectral function, *A*(**k**, *ω*) and *A*(*ω*), that can be directly compared, respectively, with the DFT-calculated band structure and DOS. We see an upshift of the *Γ* pocket above the Fermi energy also for *n* = 7 in DMFT. For *n* = 4 now also the A pocket is shifted above the Fermi level, while it still crosses the Fermi energy for *n* = 7. Only the Ni ${d}_{{x}^{2}-{y}^{2}}$ orbital that crosses the Fermi level has a strong quasiparticle renormalization *Z* = 0.32 for *n* = 4 and *Z* = 0.26 for *n* = 7, which also causes a finite-life-time broadening compared to the bare DFT dispersion. The renormalizations *Z* of all other orbitals remain close to one, see Supplementary Note 4. Further, we see some broadened lower Hubbard band around −0.8 eV which comprises all Ni *d* orbitals.

For determining the proper number of electrons in the Ni ${d}_{{x}^{2}-{y}^{2}}$ orbital, the filling of the A pocket needs to be taken into account. In the Supplementary Note 5, we determine the DMFT hole doping due to the A pocket to be roughly *δ*_pocket_ ≈ 0.02 − 0.08 × 1/*n* for *n* > 4 and zero for *n* ≤ 4 where there is no A pocket.

**Fig. 4:**
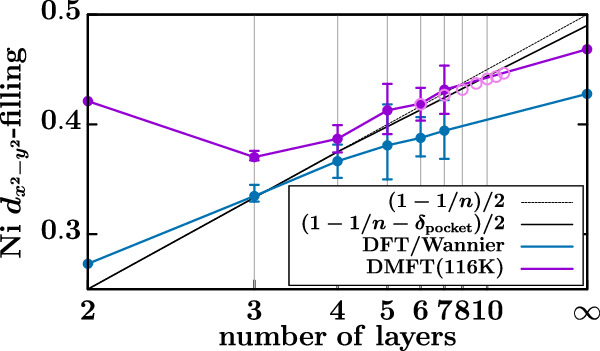
Average Ni ${d}_{{x}^{2}-{y}^{2}}$ occupation as a function of the number of layers *n.* Compared are DMFT at 116 K (which is almost identical to 300 K) to DFT to the formal valence count with and without including the self-doping through the A pocket. Open circles for *n* ≥ 6 indicate, as in Fig. 1, an infinite-layer calculation with hole doping 1/*n*.

Figure 4 compares the filling of the Ni ${d}_{{x}^{2}-{y}^{2}}$ orbital with this hole doping (1 − 1/*n* − *δ*_pocket_)/2 to the DMFT occupation of the ${d}_{{x}^{2}-{y}^{2}}$ orbital, where the factor 1/2 accounts for the spin. For larger *n* both well agree, though the infinite-layer compound somewhat deviates from our *δ*_pocket_ fitted to finite-layer nickelates. For *n* ≲ 4, on the other hand, we see deviations in DMFT. The reason for these deviations is two-fold: (i) There is some, actually quite small admixture of the Ni ${d}_{{x}^{2}-{y}^{2}}$ orbital with the other Ni and Nd orbitals. Both give opposite effects for the filling of Ni ${d}_{{x}^{2}-{y}^{2}}$ orbital and essentially cancel for *n* > 4. When the number of layers is smaller this is not the case any more because the Nd layers adjacent to the stacking faults do not contribute in this admixture, see Supplementary Note 4 for a detailed discussion. (ii) For *n* = 2, the Ni ${d}_{{z}^{2}}$ orbital crosses the Fermi level in DMFT (but not in DFT; see Supplementary Note 4). Its depopulation allows the Ni ${d}_{{x}^{2}-{y}^{2}}$ orbital to stay closer to half-filling.

This also means that for *n* = 2 we have multi-orbital physics involving both Ni ${d}_{{z}^{2}}$ and ${d}_{{x}^{2}-{y}^{2}}$ similar as for the Ruddlesden-Popper nickelate La_3_Ni_2_O_7_^17^. For *n* > 2, on the other hand, there is neither a Ni ${d}_{{z}^{2}}$ Fermi surface nor a *Γ* pocket in DMFT, only a Nd-derived A pocket, in agreement with ARPES experiments for the infinite-layer limit *n* → *∞*^36,37^.

**Fig. 5:**
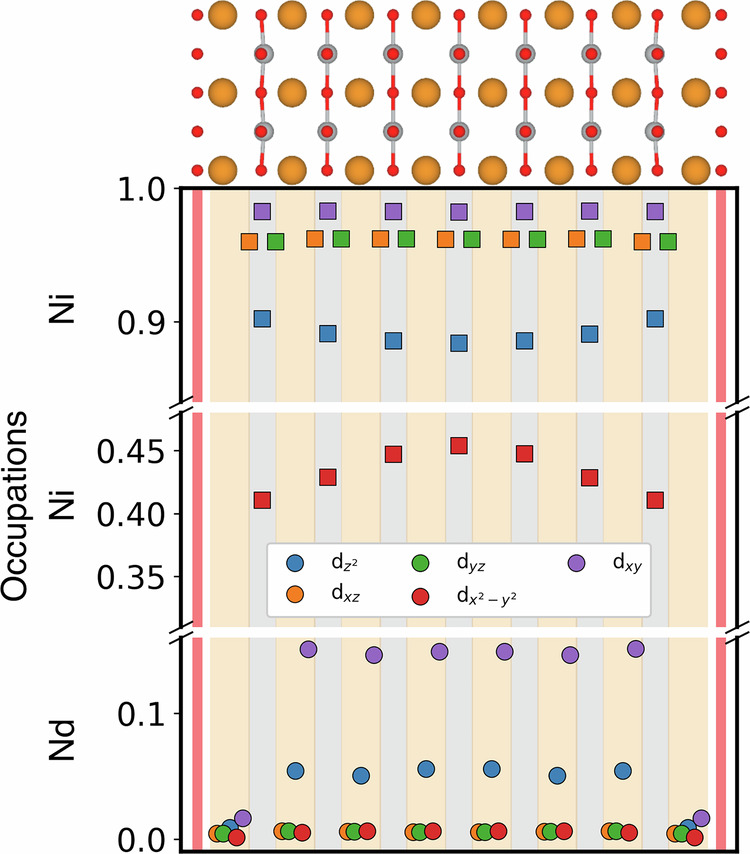
DMFT orbital occupations for each Ni and Nd atom of Nd_8_Ni_7_O_16_. See Supplementary Note 4 for other *n*. The top of the figure shows the crystal structure with Nd, Ni and O atoms displayed in orange, gray, and red, respectively. The same color code is used in the bottom part for the background stripes to visualize the Nd, Ni(O_2_), and stacking-fault O planes. Orbital occupations of Ni and Nd are displayed in their respective stripe and represented with squares and circles, respectively, with the color code for the different 3*d* respectively 5*d* orbitals given in the legend box. Note the broken *y*-axis with the almost empty Nd, half-filled Ni ${d}_{{x}^{2}-{y}^{2}}$, and nearly occupied other Ni orbitals.

Figure 5 further shows the layer- and orbital-resolved DMFT occupation for *n* = 7, with the aforementioned orbital admixture leading to a Ni ${d}_{{z}^{2}}$ occupation around 0.9 instead of 1, some Nd ${d}_{{z}^{2}}$ occupation and a Nd *d*_*xy*_ occupation larger than what to expected from the size of the A pocket alone. Most importantly, however, for the Ni ${d}_{{x}^{2}-{y}^{2}}$ orbital we observe a quite strong layer dependence. There are sizably more holes in the layers adjacent to the extra O at the stacking fault (red layer) that is responsible for the hole doping in the first place.

### Dynamical vertex approximation

By construction, DMFT accounts only for local correlations. To include non-local correlations and calculate the superconducting *T*_*c*_, the Hamiltonian with *n* × 5 Ni 3*d* and (*n* + 1) × 5 Nd 5*d* orbitals is way beyond the current technical limitations. However, the DMFT clearly shows the prevalence of the Ni 3${d}_{{x}^{2}-{y}^{2}}$ orbital; and the Nd-derived band(s) crossing the Fermi level around A (and M), do not hybridize in the bulk with the Ni 3${d}_{{x}^{2}-{y}^{2}}$ orbital. Also for the finite-layer nickelates this hybridization is weak. Hence, we describe the superconducting properties of the compound as in infinite-layer nickelates^34^, i.e., we restrict ourselves to a single 3${d}_{{x}^{2}-{y}^{2}}$ orbital per NiO_2_ layer and do an additional Wannier projection onto this single orbital. The hopping parameters of this one-Ni-${d}_{{x}^{2}-{y}^{2}}$-orbital model are given in the Supplementary Note 2; and the Hubbard interaction *U* for this single orbital must be somewhat smaller due to additional screening. We use the same cRPA-estimated *U* = 8*t* as in Ref. ^34^.

Given the rather weak inter-layer hopping *t*_*z*_, we then solve a two-dimensional Hubbard model with D*Γ*A^34,46–48^. We find *d*-wave superconductivity induced by antiferromagnetic spin fluctuations and the phase diagram has been already presented in Fig. 1. As different layers exhibit slightly different hoppings and dopings, see Fig. 4, we give – as a fair estimate of the error bar – the minimal and maximal *T*_*c*_ for three dopings: average DMFT ${d}_{{x}^{2}-{y}^{2}}$ occupation; layer with optimal DMFT ${d}_{{x}^{2}-{y}^{2}}$ occupation; and (1 − 1/*n* − *δ*_pocket_)/2. The in-plane hopping parameters are quite constant for all *n* and all layers, see Supplementary Note 2, and well described by *t* = 420 meV, $t^{\prime} =-0.25t$, *t″* = 0.12*t*.

## Discussion

Using state-of-the-art DFT, DMFT, and D*Γ*A we have calculated the superconducting phase diagram of finite-layer nickelates Nd_*n*+1_Ni_*n*_O_2*n*+2_ on a NdGaO_3_ substrate. In agreement with preliminary experimental results^18^, we obtain superconductivity for *n* ≈ 4–9 layers, where we have mimicked *n* ≥ 8 by a hole-doped infinite-layer Wannier Hamiltonian. Compared to the first infinite-layer experiments,^6,18^ the calculated *T*_c_ values are somewhat higher. However, the timeline of reported *T*_c_ in infinite-layer nickelates makes us hopeful that further experiments and improved synthesis will enhance *T*_c_ also in finite-layer nickelates.

Only the Ni ${d}_{{x}^{2}-{y}^{2}}$ orbital and the A pocket cross the Fermi energy. A somewhat unexpected result is that the hopping parameters for the Ni ${d}_{{x}^{2}-{y}^{2}}$ orbital do *not* depend on the layer index nor on the number of layers *n*. This is a consequence of growing the nickelates on a NdGaO_3_ substrate, thus fixing the in-plane lattice constant, and the tilting at the stacking fault is, in hindsight, not large enough for changing the hopping parameters substantially. This practical universality of the hopping parameters is very different from the cuprates that are synthesized as bulk materials and are thus much more versatile in their in-plane lattice constant.

However, there is quite a strong layer dependence of the Ni ${d}_{{x}^{2}-{y}^{2}}$ orbital occupation. This is relevant for the superconducting *T*_*c*_. Within the constraints of what is presently feasible, we take this layer-dependent doping into account by calculating *T*_*c*_ in D*Γ*A for a two-dimensional single-${d}_{{x}^{2}-{y}^{2}}$-orbital Hubbard model, with the error bars in Fig. 1 reflecting in particular the uncertainties and layer-dependencies of the doping of the Ni ${d}_{{x}^{2}-{y}^{2}}$ orbital. This uncertainty results in a particular large error bar for *n* = 4. We feel that, nonetheless and with this caveat in mind, our calculation of the phase diagram is accurate and reliable, without any adjusted parameters.

## Methods

In this section, we summarize the computational methods employed. The interested reader can find additional information in^43^ (including its Supplementary Information) and^34,49^; data and input files for the whole set of calculations in the associated data repository 10.48436/d608p-bba49.

Density functional theory calculations were performed using the Vienna ab-initio simulation package (VASP)^50,51^ using projector-augmented wave pseudopotentials and Perdew-Burke-Ernzerhof exchange correlation functional adapted for solids (PBESol)^52,53^, with a cutoff of 1000 eV for the plane wave expansion. Integration over the Brillouin zone was performed over a grid with a uniform spacing of 0.25 Å^−1^ and a Gaussian smearing of 0.20 eV. Wannierization was performed using wannier90^54^.

The crystal structures structures were relaxed first by fixing the in-plane lattice parameter to the NdGaO_3_ value (reference substrate, 3.83 *Å*). Then the internal coordinates were relaxed along with the *c* axis as in ref. ^43^.

DMFT calculations were performed using w2dynamics^55^ using continuous-time quantum Monte Carlo simulations in the hybridization expansion (CTHYB) as an impurity solver^56,57^, a Kanamori interaction with the values of *U* and *J* as detailed in the main text has been taken at room temperature (300 K) and 116 K (corresponding to 0.01 eV). The fully localized limit was used as double-counting scheme, as introduced in^58^. The self-energies were continued from the Matsubara to the real-frequency axis using the package ana_cont and the “chi2kink” method as described in ref. ^59^.

For obtaining *T*_c_, we employed the ladder D*Γ*A with the *λ* correction as done before in refs. ^34,60–62^. To evaluate *T*_c_ of the target fillings for drawing the phase diagram, we interpolated (extrapolated) the D*Γ*A results for Ni ${d}_{{x}^{2}-{y}^{2}}$ fillings *n* = 0.775, 0.80, 0.825, 0.85, 0.875, 0.90, which were obtained from ref. ^34^ (See Supplementary Note 6 for more details).

## Supplementary information

Supplementary information

## Data Availability

The raw data for the figures reported, along with input and output files, is available at the TU Wien repository 10.48436/d608p-bba49.
