# Supplementary material for: Superconducting phase diagram of finite-layer nickelates Ndn+1NinO2n+2
Source: NPJ Quantum Mater. 2025 Jul 1;10(1):69. doi: 10.1038/s41535-025-00786-z (PMC12213617; doi:10.1038/s41535-025-00786-z)
Supplement: Supplementary file 1 — Supplementary information [file 41535_2025_786_MOESM1_ESM.pdf]

Supplementary Information: Superconducting phase diagram of finite-layer nickelates $Nd_{n+1}Ni_nO_{2n+2}$

Andreas Hausoel ,[1,*] Simone Di Cataldo ,[2,*] Motoharu Kitatani ,[3] Oleg Janson ,[1] and Karsten Held [4]

[1] *Institute for Theoretical Solid State Physics, Leibniz Institute for Solid State and Materials Research Dresden, Helmholtzstr. 20, 01069 Dresden, Germany*
[2] *Dipartimento di Fisica, Sapienza Università di Roma, Piazzale Aldo Moro 5, 00187 Roma, Italy*
[3] *Department of Material Science, University of Hyogo, Ako, Hyogo 678-1297, Japan*
[4] *Institute of Solid State Physics, TU Wien, 1040 Vienna, Austria*
(Dated: April 2, 2025)

In Supplemental Note 1, we present some details on the structural relaxation for the finite-layer nickleates and quantify the deviation from a planar Ni-O-Ni angle in the NiO_2 layers. In Supplemental Note 2, details of the Wannier projection and the tight-binding parameters for the single Ni $3d_{xy}$ orbital are given. In Supplemental Note 3 and Supplemental Note 4 the density functional theory (DFT) and dynamical mean-field theory (DMFT) results of the main text is supplemented by the same figures for the other numbers of layers n considered. In Supplemental Note 5 we calculate the number of electrons in the DMFT A pockets. Finally, in Supplemental Note 6, we briefly recapitulate the formalism for determining T_c using the dynamical vertex approximation (DΓA).

SUPPLEMENTARY NOTE 1: CRYSTAL STRUCTURES

A. Details on structural relaxation

The ground-state structures of $Nd_{n+1}Ni_nO_{2n+2}$ for the various number of layers $n = 2 - 7$ and ∞ is obtained using the conventional cell. Here, the in-plane lattice parameter is constrained to the one for the NGO substrate, i.e. $a = b = 3.83$Å. Then, the out-of-plane lattice parameter (c) is varied in a range of ±5Å around an initial guessed value of $\frac{\sqrt{3}}{2}(n+1)$, where n is the number of layers. For each value of c, the atomic positions is relaxed while the cell is kept fixed. The resulting curve of enthalpy vs c value is then fitted with a fourth-degree polynomial to determine the minimum, which was considered the relaxed value for the c axis. The values of c obtained in this way are then employed to construct the primitive cell. We note that the trend of c axis against n is perfectly linear, with $c = (6.56 \cdot n + 5.80)$Å

n	c (Å)
2	18.9
3	25.5
4	32.1
5	38.7
6	45.2
7	51.7

Supplementary Table S1. c axis values as a function of n obtained from the DFT relaxation.

There is considerable Ni-O-Ni out-of-plane distortion, visible with the naked eye in Fig. 1 (top) of the main text. In Fig. S1 we here additionally report the actual angle of this distortion for $n = 2 \ldots 7$. There is a large distortion at the stacking fault (the first and last layer), while the central layers are almost planar.

---

[*] These authors contributed equally.

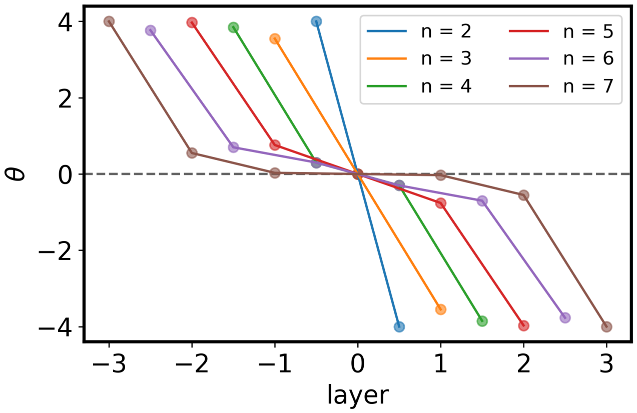

Supplementary Figure S1. Angle formed by the Ni–O bond with respect to the xy plane as a function of the layer number for finite-layer nickelates with $n = 2 - 7$ layers in total. The x axis indicates the layer number, with zero corresponding to the innermost layer.

SUPPLEMENTARY NOTE 2: WANNIER PROJECTION AND TIGHT-BINDING PARAMETERS

Maximally localized Wannier functions are obtained from the DFT band structure calculated using VASP [1] following the Marzari-Vanderbilt procedure as implemented in Wannier90 [2]. The non-self-consistent calculation of the Kohn-Sham eigenvalues is performed over an $8 \times 8 \times 8$ grid, with an energy cutoff of 1000 eV on the plane waves expansion. The disentanglement of the Wannierization is carried out with the energy windows specified in Tab. S2, with a convergence tolerance of 10^{-10}Å^2 or up to 4200 iterations, with a mix ratio of 0.200. Minimization of the spread is carried out for 1200 iterations. In all cases we find that the spread of the Wannier functions is never larger than 3.0 Å^2. In Table S2, we report in detail the outer energy window, the frozen energy window, and the initial projections, for each of the n layers.

number of layers	`dis_win_min`	`dis_win_max`	`froz_win_min`	`froz_win_max`	`begin_projections`
Ni d + Nd d; 10×n-bands wannierization					
n = 2	-4.50	10.80	-1.19	2.61	Ni:d, Nd:d
n = 3	-4.87	10.13	-1.66	2.14	Ni:d, Nd:d
n = 4	-4.40	9.20	-1.89	1.71	Ni:d, Nd:d
n = 5	-6.18	9.72	-1.87	1.53	Ni:d, Nd:d
n = 6	-4.73	10.57	-2.22	0.58	Ni:d, Nd:d
n = 7	-3.77	10.53	-1.26	1.54	Ni:d, Nd:d
Ni - $d_{x^2-y^2}$; 1×n-bands wannierization					
n = 2	-1.13	2.97	-0.13	0.37	Ni:$d_{x^2-y^2}$
n = 3	-1.40	2.73	-	-	Ni:$d_{x^2-y^2}$
n = 4	-1.63	2.67	-	-	Ni:$d_{x^2-y^2}$
n = 5	-1.37	2.92	-	-	Ni:$d_{x^2-y^2}$
n = 6	-1.32	2.58	-	-	Ni:$d_{x^2-y^2}$
n = 7	-2.49	4.01	-	-	Ni:$d_{x^2-y^2}$

Supplementary Table S2. Summary of the main Wannierization parameters for the various number of layers n. **Note:** to avoid machine or pseudopotential dependency, all the energy values are given with respect to the Fermi energy.

In Tables S3 to S8 we report the hopping parameters for the Wannier projection onto a single Ni $3d_{xy}$ orbital for the $Nd_{n+1}Ni_nO_{2n+2}$ series, with n going from 2 to 7. Here, t is the nearest neighbor hopping, t' the diagonal next-nearest neighbor hopping , t'' the next-next-neighbor hopping with two-site distance along the x (or y) direction, and t_z nearest neighbor hopping in the z-direction between the NiO_2 planes.

n = 2	t (eV)	t' (eV)	t'' (eV)	t'/t	t''/t	t_z (eV)
	-0.41	0.10	-0.06	-0.24	0.14	-0.06
	-0.41	0.10	-0.06	-0.24	0.14	

Supplementary Table S3. Hopping parameters for $n = 2$. The hoppings are listed in the order of the layers (from top to bottom). The interlayer hopping t_z between Ni-d$_{x^2-y^2}$ orbitals in adjacent layers is shown between one layer and the next.

n = 3	t (eV)	t' (eV)	t'' (eV)	t'/t	t''/t	t_z (eV)
	-0.42	0.10	-0.05	-0.24	0.12	-0.03
	-0.42	0.10	-0.05	-0.25	0.12	-0.03
	-0.42	0.10	-0.05	-0.24	0.12	

Supplementary Table S4. As in Table S4 but for $n = 3$.

n = 4	t (eV)	t' (eV)	t'' (eV)	t'/t	t''/t	t_z (eV)
	-0.41	0.10	-0.05	-0.24	0.13	-0.03
	-0.42	0.10	-0.05	-0.25	0.13	-0.03
	-0.42	0.10	-0.05	-0.25	0.13	-0.03
	-0.41	0.10	-0.05	-0.24	0.13	

Supplementary Table S5. As in Table S4 but for $n = 4$.

n = 5	t (eV)	t' (eV)	t'' (eV)	t'/t	t''/t	t_z (eV)
	-0.41	0.10	-0.05	-0.24	0.13	-0.03
	-0.41	0.10	-0.05	-0.25	0.12	-0.03
	-0.41	0.11	-0.05	-0.25	0.12	-0.03
	-0.41	0.10	-0.05	-0.25	0.12	-0.03
	-0.41	0.10	-0.05	-0.24	0.13	

Supplementary Table S6. As in Table S4 but for $n = 5$.

n = 6	t (eV)	t' (eV)	t'' (eV)	t'/t	t''/t	t_z (eV)
	-0.41	0.10	-0.05	-0.25	0.13	-0.03
	-0.42	0.10	-0.05	-0.24	0.13	-0.03
	-0.41	0.10	-0.05	-0.25	0.13	-0.03
	-0.41	0.10	-0.05	-0.25	0.13	-0.03
	-0.42	0.10	-0.05	-0.24	0.13	-0.03
	-0.41	0.10	-0.05	-0.25	0.13	

Supplementary Table S7. As in Table S4 but for $n = 6$.

n = 7	t (eV)	t' (eV)	t'' (eV)	t'/t	t''/t	t_z (eV)
	-0.41	0.10	-0.05	-0.24	0.12	-0.02
	-0.41	0.10	-0.05	-0.24	0.12	-0.03
	-0.41	0.10	-0.05	-0.25	0.12	-0.03
	-0.41	0.10	-0.05	-0.25	0.12	-0.03
	-0.41	0.10	-0.05	-0.25	0.12	-0.03
	-0.41	0.10	-0.05	-0.24	0.12	-0.02
	-0.41	0.10	-0.05	-0.24	0.12	

Supplementary Table S8. As in Table S4 but for $n = 7$.

SUPPLEMENTARY NOTE 3: ADDITIONAL DFT RESULTS

In this Supplemental Note, we present in Fig. S2 the DFT band-structure and density of states (DOS) also for $n = 2$, $n = 3$, $n = 5$ and $n = 6$. The DFT results for $n = 4$ and $n = 7$, already presented in Fig. 2 of the main text, are repeated for convenience.

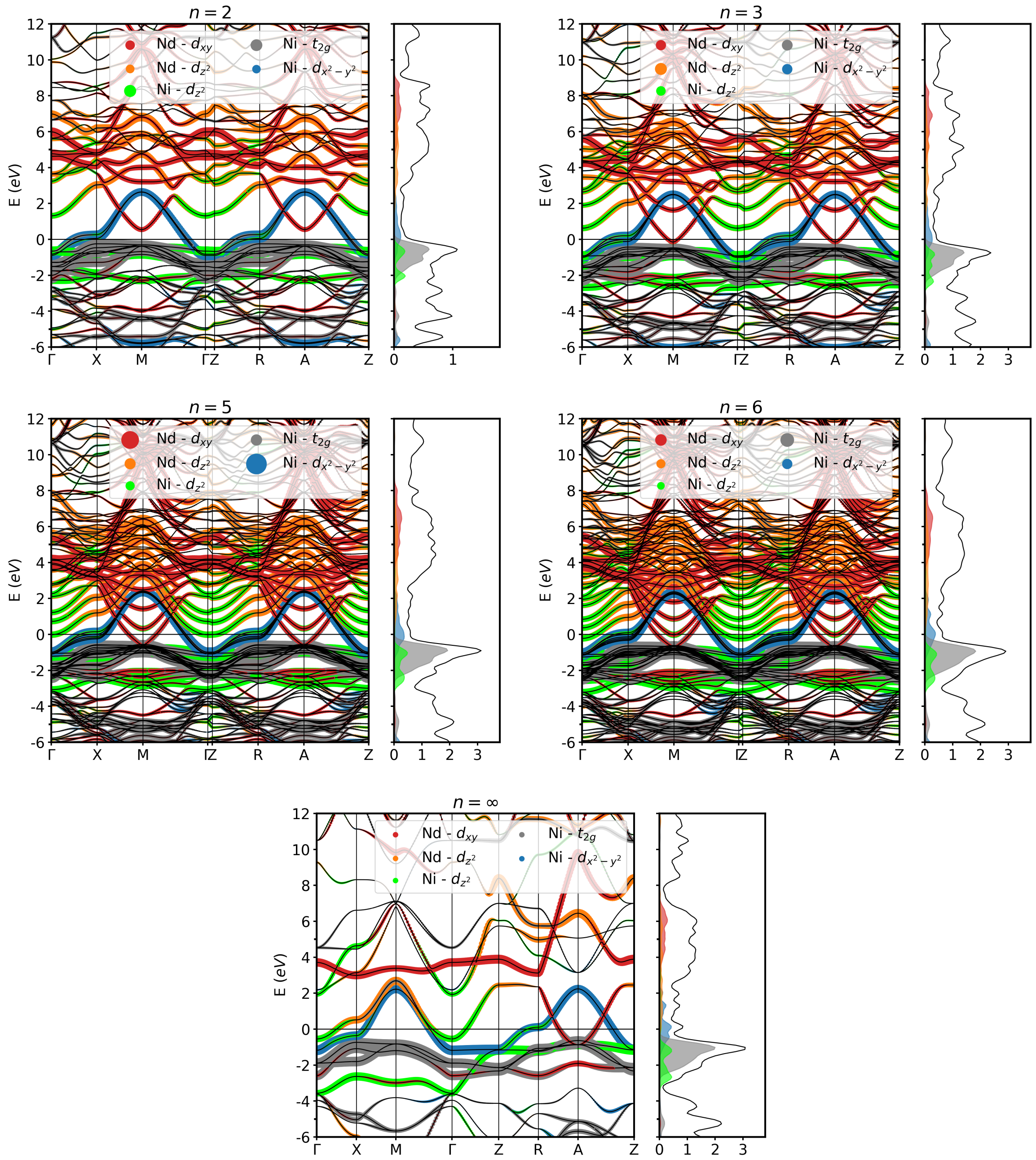

Supplementary Figure S2. DFT band structure for finite-layer nickelates $Nd_{n+1}Ni_nO_{2n+2}$ ($n = 2, 3, 5, 6, \infty$). The Fermi energy is taken as the energy zero. Projections onto different atomic orbitals are shown as thick colored lines, where thickness indicates the strength of the projection.

Further, in Fig. S3 we show the DFT Fermi surface for the finite-layer nickelates considered with $n = 2 \ldots 7$ layers, as well as for the infinite-layer nickelates. With the stacking fault well separating each n layers from the next n, there is essentially no k_z dispersion any more. Instead, we get n copies of the Ni $3d_{xy}$ and n+1 copies of the Nd d_{xy} orbital which forms the pocket around the A pocket momentum (see the $k_z = \pi$ plane) for the infinite-layer nickelate. For

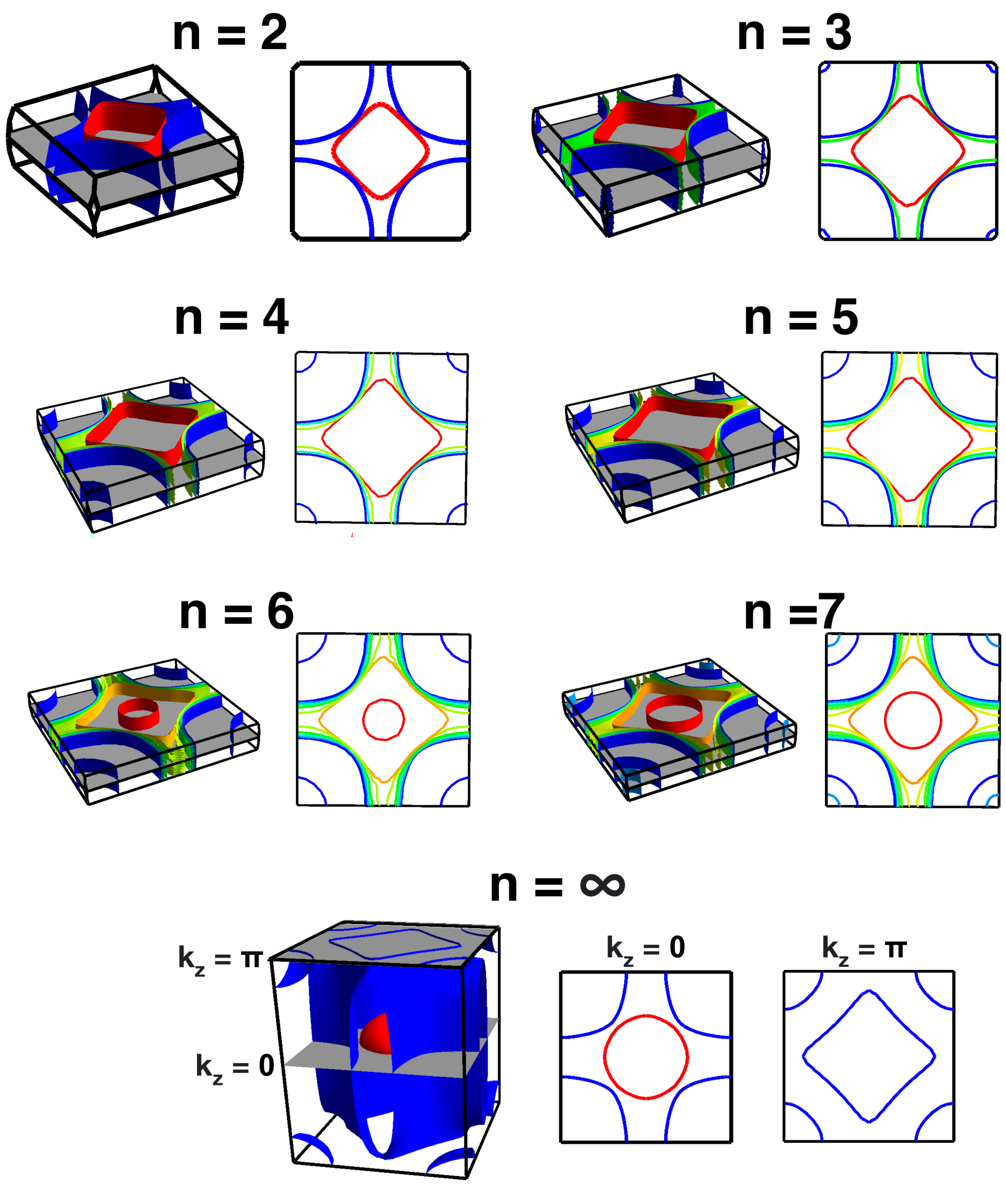

Supplementary Figure S3. DFT-calculated Fermi surfaces for different number of layers n.

the finite-layer nickelates, this pocket just emerges for $n = 2$ as a tube instead of a pocket, comprising the A and M point. For $n = 7$ a second tube emerges.

As for the Ni $3d_{xy}$ Fermi surface, we have for the infinite-layer case in Fig. S3, a hole-like Fermi surface for $k_z = 0$ and an electron-like for $k_z = \pi$. Concomitantly, we have both hole-like and electron-like Fermi surfaces for finite n, with the second electron-like Fermi surface appearing for $n = 5$.

Further, in Fig. S4 we show the layer-resolved bandstructure and partial DOS. The overall picture is that the stacking fault perturbs particularly the z direction for the outer layers. This is quite intuitive if one considers that it is only in that direction that the stacking fault breaks symmetry and the outer layers are most affected. The effect on Ni $3d_{x^2-y^2}$ is that the inner layers have more weights on the outer two bands in the four-band strip between Γ and X. In the DOS this results in a split of the van Hove peak into two peaks just above and below the Fermi energy.

In Fig. S5 we also show the DFT band structure for a heterostructure comprising $n = 3$ and $n = 4$ slabs, with orbital projections resolved with respect to both atomic orbitals and slab. One can see that the bands of different slabs are "independent", i.e. there are no mixed partial projections for the two slabs.

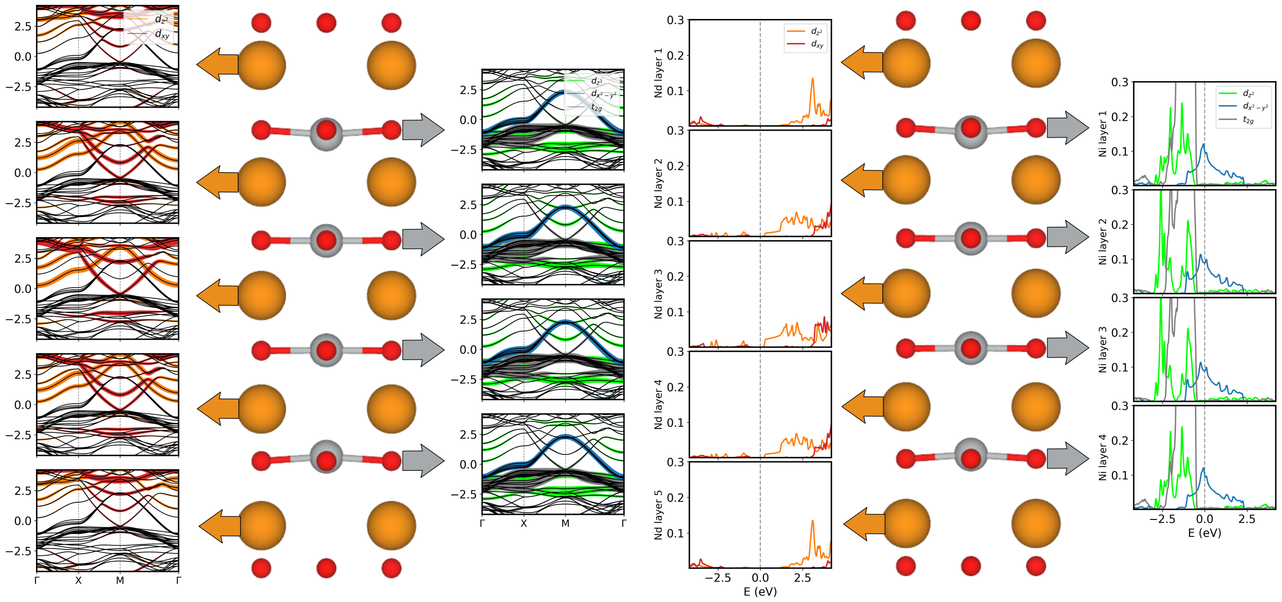

Supplementary Figure S4. Layer-resolved band structures (left) and partial DOS (right) for $n = 4$.

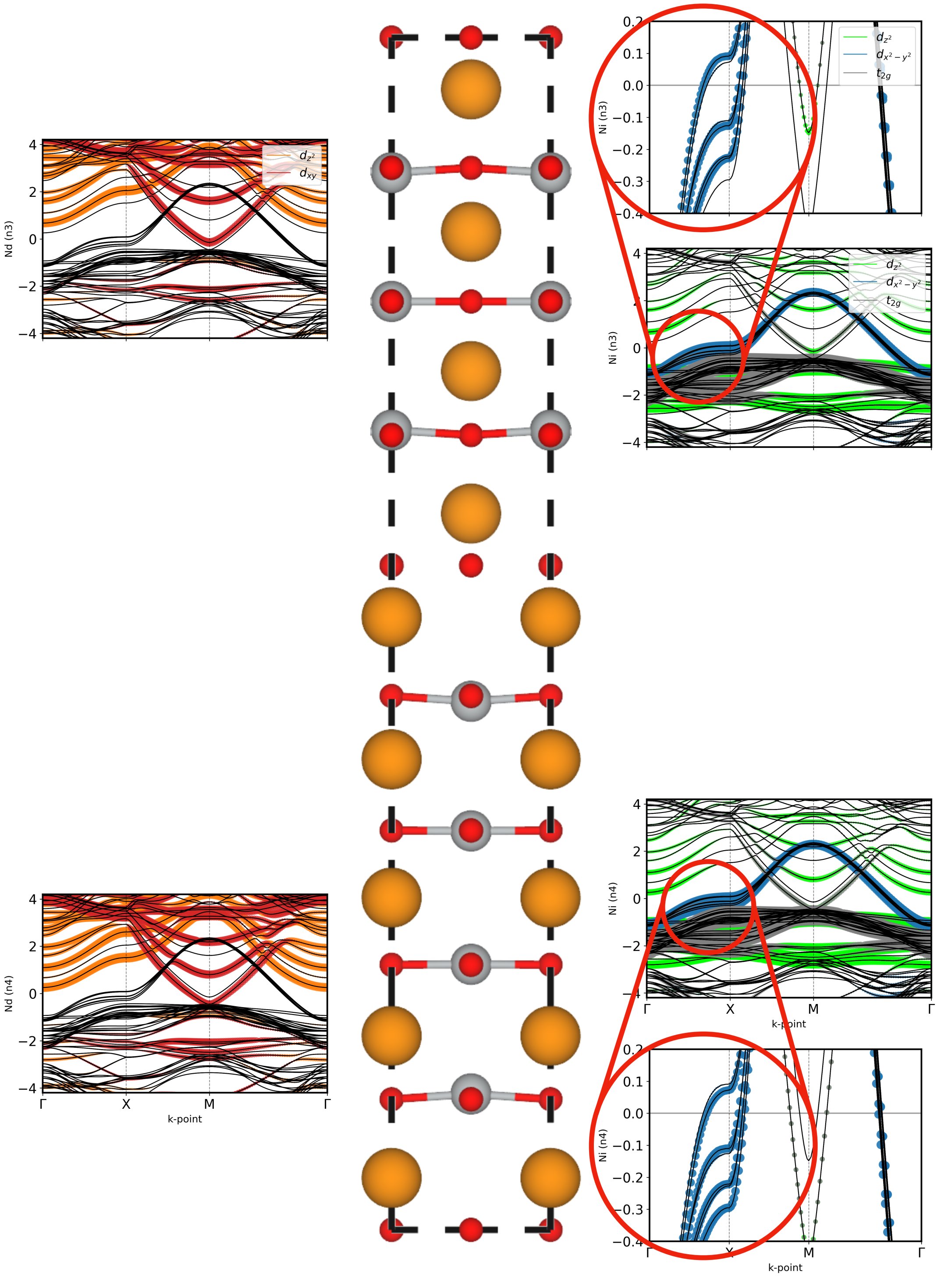

Supplementary Figure S5. DFT band structure of a "$n = 3$" + "$n = 4$" heterostructure with slab- and orbital-resolved projection (fat blue bands). Top: projection on the first $n = 3$ slab; bottom: $n = 4$ slab for Nd orbitals (left) and Ni orbitals (right; including a zoom-in).

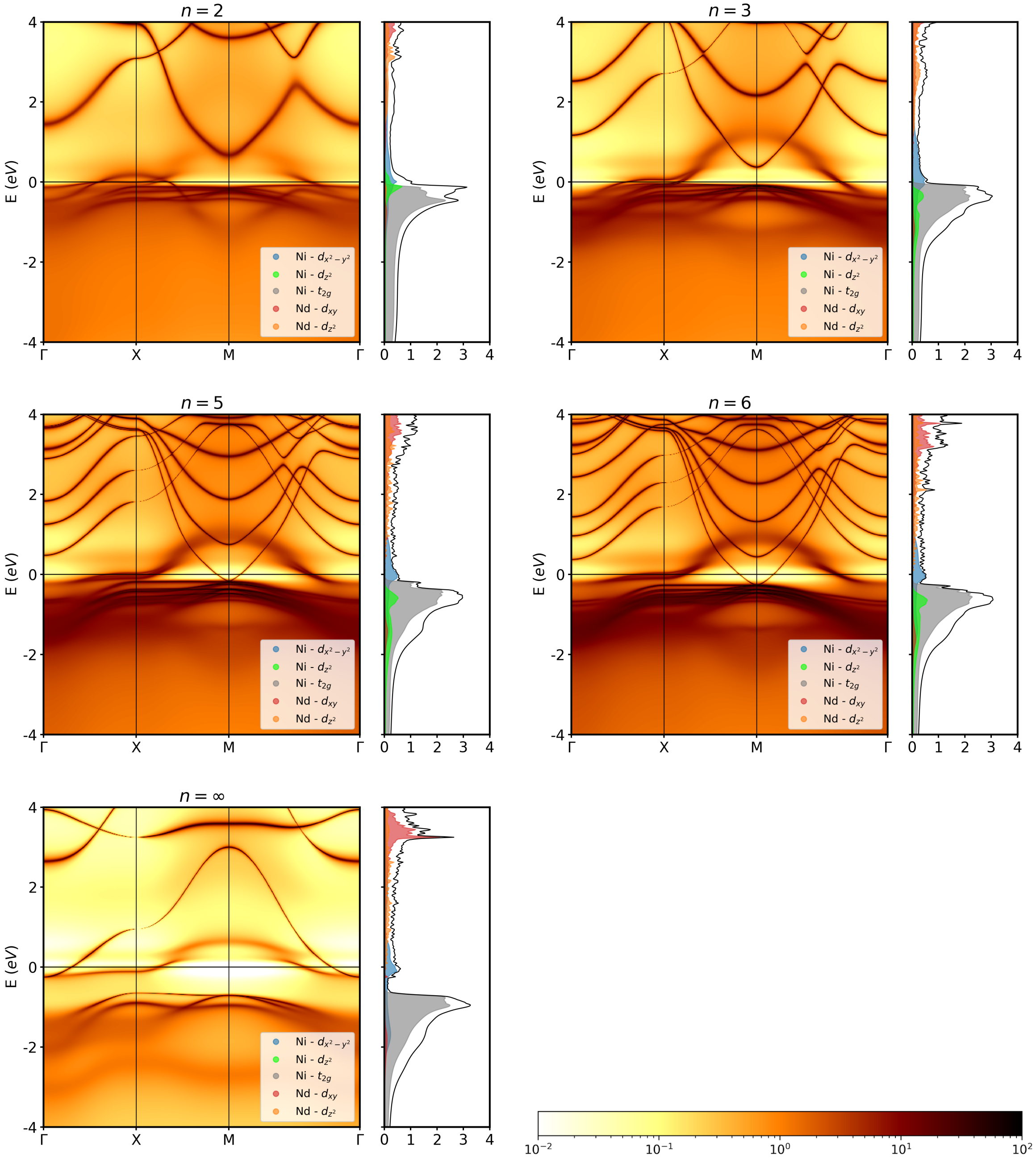

Supplementary Figure S6. DMFT electronic structure and DOS of $Nd_{n+1}Ni_nO_{2n+2}$ for $n = 2, 3, 5, 6, \infty$ at room temperature.

SUPPLEMENTARY NOTE 4: ADDITIONAL DMFT RESULTS

In Fig. S6 we show, additionally to Fig. 3 of the main text, also the DMFT spectral function and DOS for $n = 2, 3, 5, 6$. For $n = 2$, the hole doping is so large that the DMFT physics turns: the Ni d_{z^2} orbital also crosses the Fermi level and we have actual multi-Ni-orbital physics. For $n > 2$ the qualitative behavior is similar, with as a matter of course more vineyard-like bands for larger n. Further we provide in Fig. S7 all calculated quasiparticle renormalizations.

In Fig. S8 we show the occupation for all atoms (layers) and all orbitals also for other n besides the $n = 7$ in Fig. 5 of the main text. Besides the DMFT results, also those of DFT, more precisely the DFT-derived Wannier Hamiltonian are shown. We notice for all n a somewhat larger DMFT occupation for the $3d_{xy}$ orbital than in DFT, but the tendency to have more holes in the layers interfacing the stacking fault is the same in DFT and DMFT. For

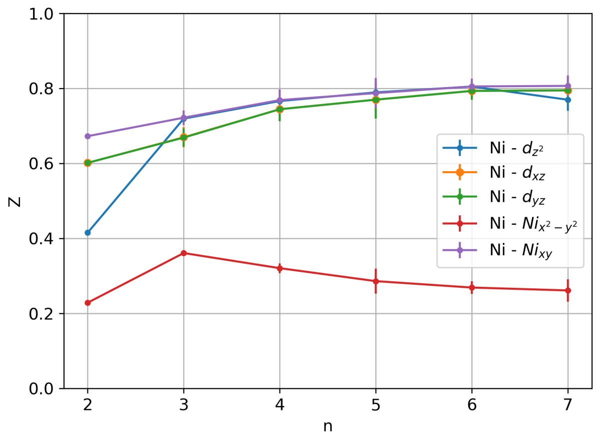

Supplementary Figure S7. Quasiparticle renormalizations of the Ni orbitals in DMFT as a function of the number of layers n at room temperature. Here, orbital 1 to 5 are the Ni z^2, xz, yz, x^2-y^2 and xy orbital, respectively. The error bar reflects the variation of Z from layer to layer.

$n=2$ the difference between DMFT and DFT occupation is strongly enhanced, as the DMFT Ni d_{z^2} orbital crosses the Fermi level.

While for $n=3\ldots\infty$ no orbital other than the predominantly Ni $3d_{xy}$ quasiparticle band and the Nd-d_{xy}-derived pocket cross the Fermi level, this orbital has some (albeit small) admixture with the Ni and Nd other orbitals. This admixture explains the deviations from the occupation $(1-1/n-\delta_{\text{pocket}})/2$ of the Ni $3d_{xy}$ band, with δ_{pocket} determined in Supplemental Note 5.

For example, in Fig. 2 of the main text or in Fig. S2 one sees that there is also a small Ni d_{z^2} contribution (green) to the predominantly Ni $3d_{xy}$ orbital at and below the X and R point. Vice versa there is a small Ni $3d_{xy}$ admixing to the other Ni bands below the Fermi energy (blue). Furhter, we observe a Ni t_{2g} admixture (grey) to the Nd d_{xy} band around A, and a somewhat larger admixture between Ni d_{z^2} and Nd d_{z^2} (green and orange).

For the orbital- and layer-resolved occupations in Fig. S8, this orbital admixture altogether leads to a Ni d_{z^2} occupation of 0.9 instead of 1, Ni $d_{xz/yz}$ of 0.97; Nd d_{z^2} of about 0.05 and Nd d_{xy} of about 0.15 (which is beyond what to expect from the A pocket size). For the nearly half-occupied Ni $3d_{xy}$ orbital these effects essentially cancel for $n>4$ in DMFT. In contrast, the DFT occupations in Fig. 4 of the main text lie below $(1-1/n-\delta_{\text{pocket}})/2$. In this respect, keep in mind though that the DFT pocket is larger than the plotted δ_{pocket} of DMFT and is already present for $n=3$. The cancellation of the admixture effects in DMFT, is no longer valid for $n\lesssim 3$. The first reason for this is that there is essentially no hybridization with the outermost Nd orbitals: in Fig. S8 all Nd orbitals of the first and last layer are unoccupied, only those in the middle have a finite occupation because of the hybridization. For $n=2$ a second major factor for the deviation from this analytical expectation is that the DMFT Ni d_{z^2} orbital now also crosses the Fermi level. It is thus much more depopulated, the Ni $d_{x^2-y^2}$ orbital vice versa closer to half-filling.

Further, in Fig. S9 and S10 we show the DMFT self-energies of the Ni and Nd orbitals, respectively, for the 7-layer system as measured directly by continuous-time quantum Monte Carlo simulations in the hybridization expansion at Matsubara frequencies $\omega_n=(2n+1)\pi T$. The slope of ImΣ for $\omega_n\to 0$ directly relates to the quasiparticle renormalization which is largest for the strongly correlated Ni $3d_{x^2-y^2}$ orbital. It is already much smaller for the other Ni orbitals; and for the Nd $5d$ orbitals ImΣ is by more than one order of magnitude smaller, reflecting the weak quasiparticle renormalization of the Nd bands.

For R$e\Sigma$, the double counting (DC) correction based on the fully localized limit [3] has been subtracted, as it roughly cancels the Hartreee term, as well as the chemical potential. The real part of the self-energy describes the shift of the bands relative to each other, with electronic correlations pushing the Nd bands somewhat up relative to the Ni ones.

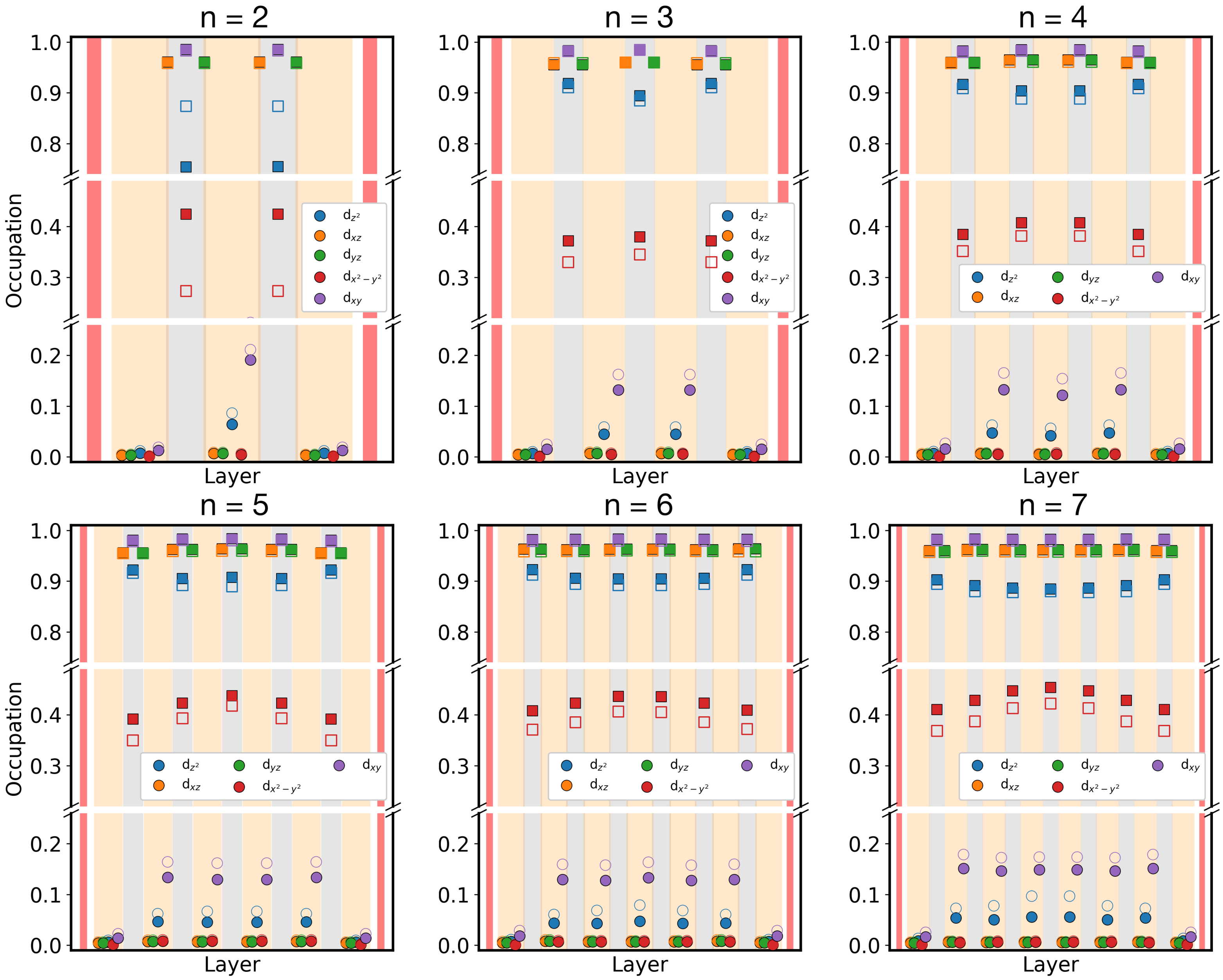

Supplementary Figure S8. Orbital- and layer-resolved Ni and Nd occupations for all the finite-layer compounds studied. The colored orange/grey/red stripes in the background represent the Nd, Ni, and defect O planes. Orbitals relative to Ni and Nd are represented respectively with squares and circles, while filled/hollow symbols indicate the DMFT and DFT (non-interacting Wannier Hamiltonian) result.

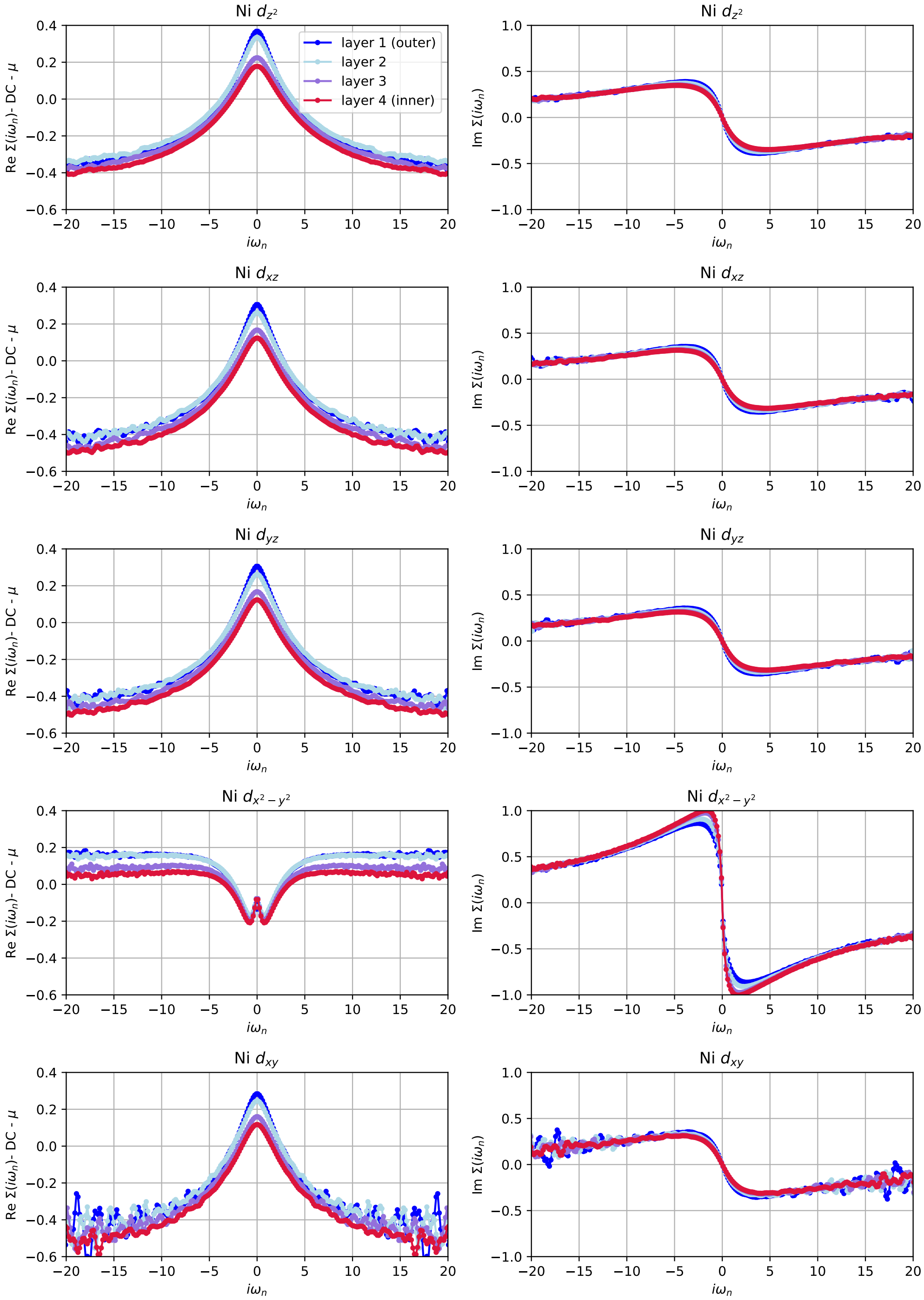

Supplementary Figure S9. Real and imaginary parts of the DMFT self-energies of the Ni 3d orbitals for the 7-layer system.

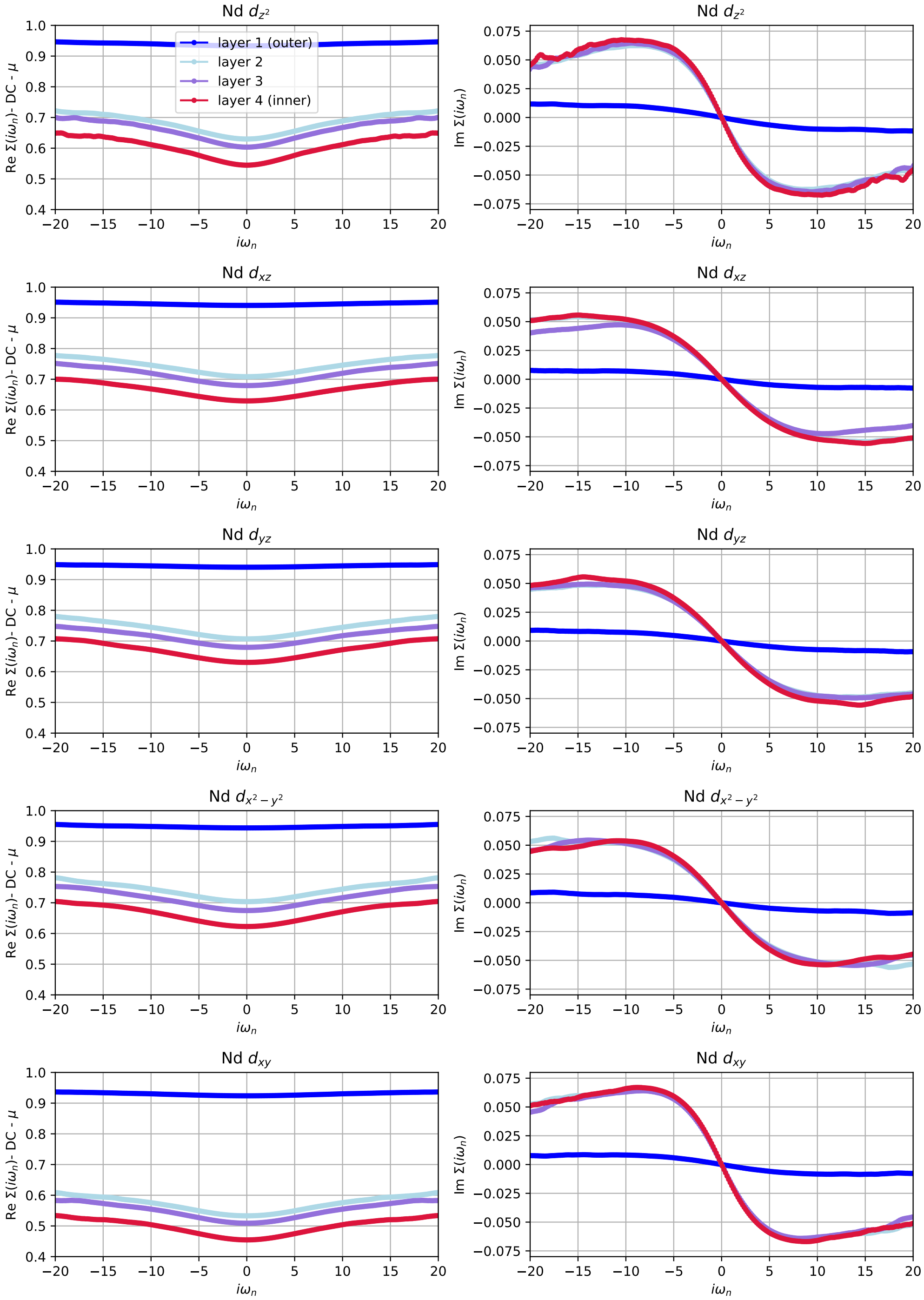

Supplementary Figure S10. Real and imaginary parts of the DMFT self-energies of the Nd 5d orbitals for the 7-layer system.

SUPPLEMENTARY NOTE 5: ELECTRONS IN THE A POCKET

In Fig. S11 we show the calculated number of electrons in the A pocket as a function of the number of layers n. The extrapolated fit (black dashed line) is (rouned to 2 digits) $\delta_{\text{pocket}} = 0.02 - 0.08 \times 1/n$. The calculation of δ_{pocket} in DMFT is actually quite involved and outlined in the following. In short, we estimate the number of electrons by the volume of a cylinder representing the Fermi surface of the A–M pocket that also in DMFT is akin to the DFT Fermi surface in Fig. S3 (just shifted).

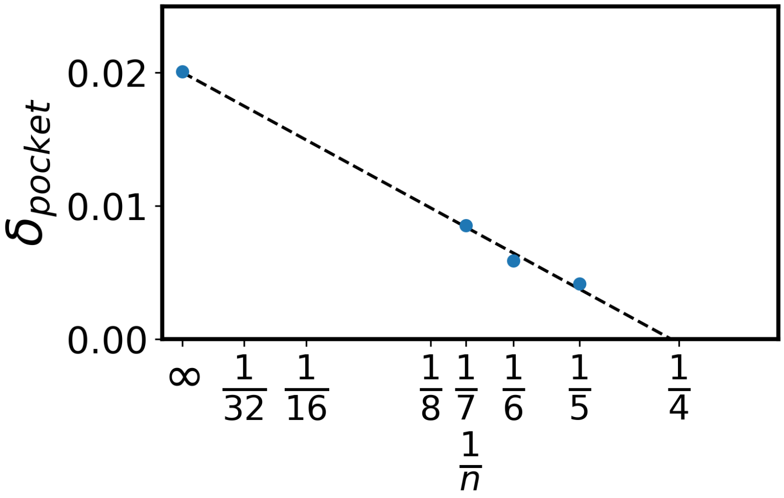

Supplementary Figure S11. Number of electrons in the A pocket as a function of the number of layers, normalized by the number of Ni atoms in the primitive cells. The black line indicates an extrapolated fit.

A. Analytic calculation

To estimate the occupation of the pocket in the A point, we proceeded as follows. First, we observe (as shown in Fig. S3) that at the DFT level the Fermi surface forming A pocket is a cylinder with its axis going from A to M, and this is particularly true for $n \geq 5$. For the infinite-layer case, the pocket is instead rather spherical.

Considering that the number of states per unit volume in the reciprocal space is constant, and that each band contains up to two electrons (counting spin), the number of occupied states in a pocket can be calculated as

$$e_p^- = 2\frac{V_p}{V_{BZ}} \tag{S1}$$

Where V_p is the volume of the pocket, and V_{BZ} is the volume of the Brillouin zone.

a. ***Ellipsoidal Fermi surface*** For a Fermi surface in the shape of an ellipsoid (quasi-spherical), the volume enclosed is

$$V_{p,s} = \frac{4}{3}\pi k_{Fx}k_{Fy}k_{Fz} \ , \tag{S2}$$

where k_{Fx}, k_{Fy}, k_{Fz} are the semi-axes of the spheroid, i.e. a sort of Fermi momentum, generalized to an ellipsoid. Substituting into Eq. (S1) we obtain that the number of states is

$$e_{p,s}^- = \frac{k_F^3 L_x L_y L_z}{3\pi^2} \ , \tag{S3}$$

where $L_x L_y L_z = V$ is the volume of the unit cell in real space.

b. ***Cylindrical Fermi surface*** For a cylindrical Fermi surface in a quasi-2D system, the volume enclosed is

$$V_{p,c} = \pi k_F^2 \frac{2\pi}{h} \tag{S4}$$

Where $\frac{2\pi}{h}$ is the height of the Brillouin zone in the shortest direction. Here k_F is to be intended as the radius of the cylinder, i.e. the Fermi momentum for the 2D case. Substituting into Eq. (S1) we obtain that the number of electrons in the A–M spheroid is

$$e^-_{p,c} = \frac{k_F^2 L_x L_y}{2\pi} , \tag{S5}$$

where $L_x L_y = A$ is the area of the top/bottom face of the unit cell in real space.

The $\mathbf{k_F}$ momentum can be identified as the $\mathbf{k}$ value for which the electron dispersion around the A pocket crosses the Fermi energy. This process must take into account the fact that electronic correlations tend to shift the pockets upwards in DMFT. To identify the momentum with the highest possible precision, we adopt the following process:

1. Superimpose the DMFT spectral function $A(\mathbf{k}, \omega)$ with the DFT bands, to find an energy shift ϵ^* such that the DFT band of the pocket corresponds to the maximum of the spectral function $A(\mathbf{k_F}, \omega = 0)$ (along the R–A line). An example for the case of $n = 5$ is shown in Fig. S12.[4]

2. Calculate the DFT bands on an extremely dense array of points between R and A

3. Shift the DFT bands by ϵ^*, and find the momentum $\mathbf{k_F}$ for which they cross the Fermi energy.

4. As A is the center of the pocket, the vector $\mathbf{\Delta k} = \mathbf{k_F} - \mathbf{A}$ has modulus equal to the radius of the cylinder (or the ellipsoid semiaxis in the R–A direction)

5. **Spheroid only:** Repeat from point 2 along the A–M direction to obtain the size of the different semiaxis in that direction.

Note: due to the crystal symmetry, the radius of the cylinder must be the same in the x and y direction.

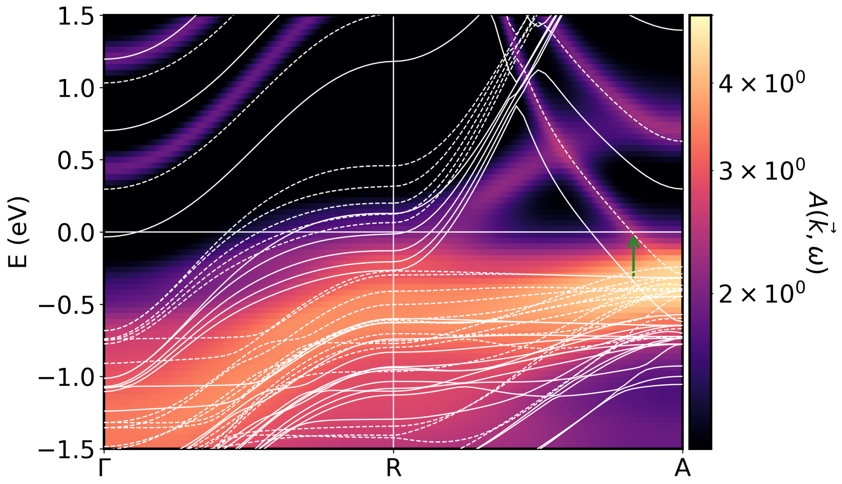

Supplementary Figure S12. Spectral function $A(\mathbf{k}, \omega)$ for $n = 5$ with DFT bands superimposed. The spectral function is indicated by the colormap, while unshifted DFT bands are shown as solid white lines, and shifted DFT bands as dashed white lines. The green arrow indicates the point in which the spectral function crosses the Fermi energy, which was used as reference to find the energy shift.

SUPPLEMENTARY NOTE 6: EVALUATION OF T_c WITH DΓA

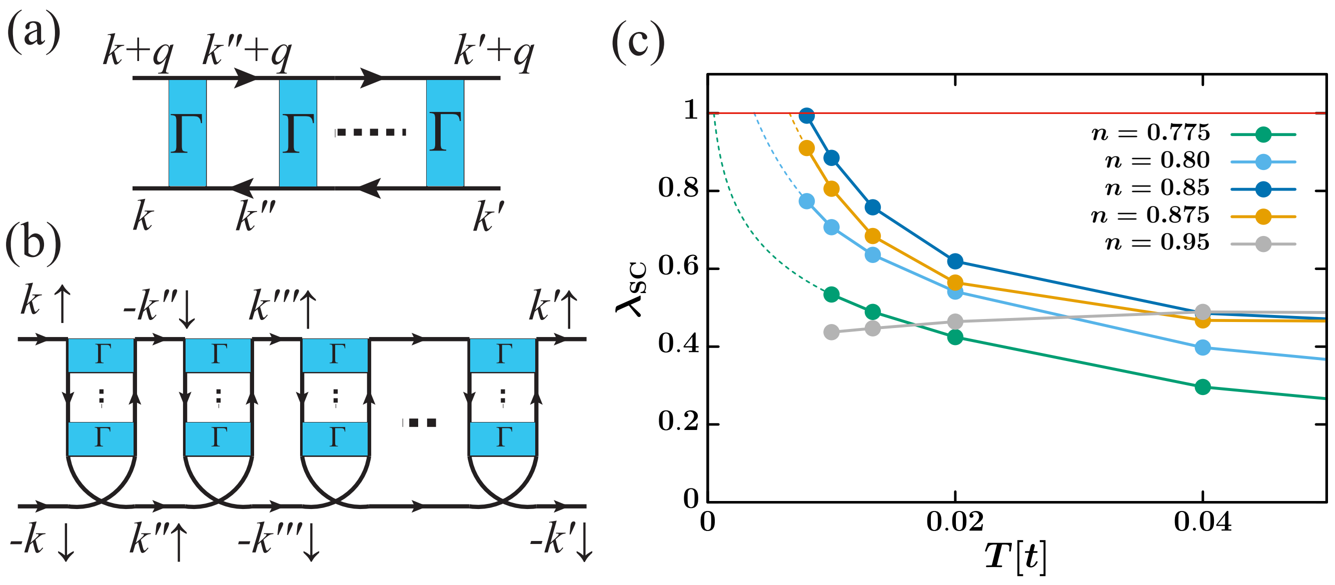

Supplementary Figure S13. (a) Diagrams represent DΓA spin fluctuations (solid line: the Green function, Γ: the irreducible vertex). (b) A particle-particle diagram represents superconducting instability mediated by DΓA spin fluctuations (an example diagram is shown). Adapted from [5, 6]. (c) The temperature dependence of the leading eigenvalue of the linearized gap equation in the square lattice Hubbard model with $t' = -0.25t, t'' = 0.12t$. Eigenvalue data taken from Ref. [7].

This section briefly reviews our formalism to determine T_c with DΓA (cf., Refs. [5, 6]). DΓA is the typical method for diagrammatic extensions of the DMFT. In this approach, we incorporate the effect of non-local spin/charge fluctuations beyond DMFT [as depicted in Fig. S13(a)] into the self-energy. After obtaining the self-energy/Green function within DΓA we further consider the particle-particle fluctuations mediated by non-local particle-hole fluctuations, as illustrated in Fig. S13(b). To analyze the divergence of this particle-particle channel, we calculate the leading eigenvalue of the linearized gap equation as

$$\lambda\Delta(k) = \frac{-1}{\beta N}\sum_{k'}\Gamma_{\mathrm{pp}}(k,k',q=0)G(k')G(-k')\Delta(k'). \tag{S6}$$

Here, G is the Green function, N is the number of k-points, Γ_{pp} is the particle-particle irreducible vertex (k, k', q are four-vectors consisting of Matsubara frequency and momentum, respectively), β is the inverse temperature, and λ (Δ) is the eigenvalue (vector) of this equation. Fig. S13(c) shows the temperature dependence of the leading eigenvalue λ of the current parameter set ($t' = -0.25t, t'' = 0.12t$). The transition temperature is determined where λ reaches the unity. From these calculations, we can obtain the relation between the filling and the transition temperature (i.e., the phase diagram) within the single-band model.

As described in the main text, we evaluated the target single-orbital fillings in multilayer systems using three different methods: (i) the average DMFT $d_{x^2-y^2}$ occupation, (ii) the layer with optimal DMFT $d_{x^2-y^2}$ occupation, and (iii) $(1 - 1/n - \delta_{\mathrm{pocket}})/2$. Then, we determined the range of T_c by comparing these fillings with the obtained phase diagram described above (The circles represent the midpoints of the determined T_c ranges).

[1] G. Kresse and J. Furthmüller, Phys. Rev. B **54**, 11169 (1996).

[2] A. A. Mostofi, J. R. Yates, Y.-S. Lee, I. Souza, D. Vanderbilt, and N. Marzari, Computer Physics Communications **178**, 685 (2008).

[3] V. I. Anisimov, J. Zaanen, and O. K. Andersen, Phys. Rev. B **44**, 943 (1991).

[4] To make sure the maximum entropy method for calculating the DMFT $A(\mathbf{k})$ is reliable, we have double checked it against a Fermi liquid fit $\mathrm{Re}\Sigma^{\mathrm{FL}}(\omega) = \mathrm{Re}\Sigma(\omega_1) + \frac{w}{w_1}\mathrm{Im}\Sigma(\omega_1)$ where ω_1 is the lowest Matsubara frequency (not shown), which confirmed the maximum entropy analytical continuation.

[5] M. Kitatani, T. Schäfer, H. Aoki, and K. Held, Phys. Rev. B **99**, 041115 (2019).
[6] M. Kitatani, R. Arita, T. Schäfer, and K. Held, Journal of Physics: Materials **5**, 034005 (2022).
[7] M. Kitatani, L. Si, O. Janson, R. Arita, Z. Zhong, and K. Held, npj Quantum Materials **5**, 59 (2020).
